# Alteration of Mevalonate Pathway in Proliferated Vascular Smooth Muscle from Diabetic Mice: Possible Role in High-Glucose-Induced Atherogenic Process

**DOI:** 10.1155/2015/379287

**Published:** 2015-03-30

**Authors:** Guo-Ping Chen, Xiao-Qin Zhang, Tao Wu, Liang Li, Jie Han, Chang-Qing Du

**Affiliations:** ^1^Department of Endocrinology, The First Affiliated Hospital, College of Medicine, Zhejiang University, Hangzhou 310003, China; ^2^Department of Respirology, Zhejiang Provincial People's Hospital, Hangzhou 310014, China; ^3^Institute of Cardiology, The First Affiliated Hospital, College of Medicine, Zhejiang University, Hangzhou 310003, China; ^4^Department of Cardiology, Zhejiang Hospital, Hangzhou 310003, China

## Abstract

The proliferation of vascular smooth muscle cells (VSMCs) is one of the main features of atherosclerosis induced by high glucose. Mevalonate pathway is an important metabolic pathway that plays a key role in multiple cellular processes. The aim of this study was to define whether the enzyme expression in mevalonate pathway is changed in proliferated VSMCs during atherogenic process in diabetic mice. Diabetes was induced in BALB/c mice with streptozotocin (STZ, 50 mg/kg/day for 5 days). Induction of diabetes with STZ was associated with an increase of lesion area and media thickness after 8 and 16 weeks of diabetes. In aorta, there were overexpressions of some enzymes, including 3-hydroxy-3-methylglutaryl-coenzyme A reductase (HMGR), farnesyl pyrophosphate synthase (FPPS), geranylgeranyl pyrophosphate synthase (GGPPS), farnesyltransferase (FNT), and geranylgeranyltransferase-1 (GGT-1), and unchanged expression of squalene synthase (SQS) and phosphor-3-hydroxy-3-methylglutaryl-coenzyme A reductase (P-HMGR) in 8 and 16 weeks of diabetes. In vitro, VSMCs were cultured and treated with different glucose concentrations for 48 h. High glucose (22.2 mM) induced VSMC proliferation and upregulation of HMGR, FPPS, GGPPS, FNT, and GGT-1 but did not change the expressions of SQS and P-HMGR. In conclusion, altered expression of several key enzymes in the mevalonate pathway may play a potential pathophysiological role in atherogenic process of diabetes macrovascular complication.

## 1. Introduction

Diabetes mellitus is a rapidly growing global problem, which is associated with increased morbidity and mortality mainly due to chronic macrovascular complications including cardiovascular disease and stroke [1, 2]. Improving macrovascular outcomes through glucose-lowering interventions has remained a difficult, complicated, and, to date, largely unsuccessful enterprise. There is an ongoing need for new therapeutic targets which would slow the development and progression of diabetic macrovascular diseases [3].

Atherosclerosis (AS) is the cause of a majority of cardio- and cerebrovascular events. Despite familiarity with this disease, some of its fundamental characteristics remain poorly recognized and understood. The proliferation of vascular smooth muscle cells (VSMCs) is one of the main features of atherosclerosis that are induced by high-glucose conditions [4]. In both type 1 and type 2 diabetes, atherosclerosis is accelerated [5]. So what is unique about proliferated vascular smooth muscle from diabetes? The answer may probably provide the next therapeutic target for macrovascular complication.

Mevalonate pathway is an important metabolic pathway which coverts mevalonate into sterol isoprenoids, such as cholesterol, and nonsterol isoprenoids, including isoprenoid intermediates-farnesyl pyrophosphate (FPP) and geranylgeranyl pyrophosphate (GGPP) [6, 7]. These nonsterol isoprenoid intermediates are essential for the posttranslational prenylation of a multitude of proteins involved in intracellular signaling (especially the small GTP-binding proteins) and are essential in cell growth/differentiation, gene expression, protein glycosylation, and cytoskeletal assembly [6, 7].

In our previous studies [8–10], we found that VSMCs from spontaneously hypertensive rats (SHR) were remarkably proliferated since 3 weeks of age; meanwhile, the expressions of key enzymes in mevalonate pathway were significantly upregulated, including 3-hydroxy-3-methylglutaryl-coenzyme A reductase (HMGR), farnesyl pyrophosphate synthase (FPPS), farnesyltransferase (FNT), and geranylgeranyltransferase-1 (GGT-1). Moreover, our team revealed that inhibition of FPPS prevented VSMCs remodeling in SHR [11]. All these data were obtained from hypertensive proliferated VSMCs. Our question then is, in diabetic proliferated VSMCs, which of the key enzymes of mevalonate pathway are altered and whether these changes are responsible for the diabetic proliferation. An earlier paper has reported that expression of GGT was significantly upregulated in brain, fat, and skeletal muscle from ob/ob diabetic mice [12]. However, detailed data about the expression of mevalonate pathway in diabetic proliferated VSMCs remain to be determined.

**Figure 1:**
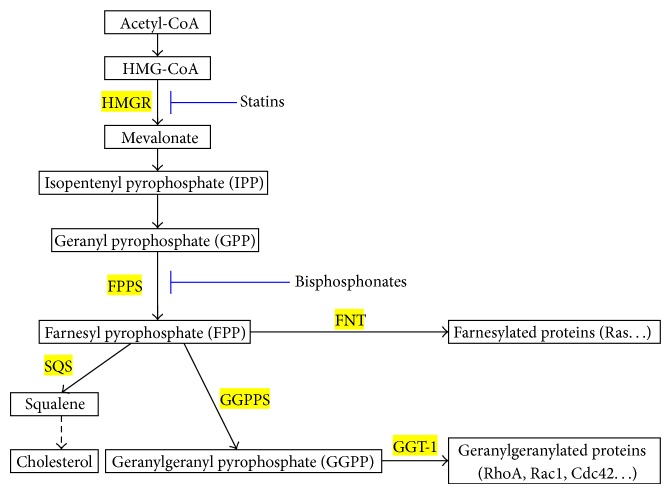
Schematic diagram of the mevalonate pathway. HMGR, 3-hydroxy-3-methylglutaryl-coenzyme A reductase; FPPS, farnesyl pyrophosphate synthase; SQS, squalene synthase; FNT, farnesyltransferase; GGT-1, geranylgeranyltransferase-1; GGPPS, geranylgeranyl pyrophosphate synthase.

Therefore, the present study was designed to investigate the expression of key enzymes of mevalonate pathway in (1) aortas from streptozotocin- (STZ-) induced 0-, 8-, and 16-week diabetic mice in vivo and (2) high-glucose-induced proliferated VSMCs in vitro, including HMGR, FPPS, FNT-*β*, GGT-1*β*, squalene synthase (SQS), phosphor-3-hydroxy-3-methylglutaryl-coenzyme A reductase (P-HMGR), and geranylgeranyl pyrophosphate synthase (GGPPS) (Figure 1).

## 2. Materials and Methods

### 2.1. Animals

Female BALB/c mice (20 ± 2.5 g) were purchased from the Shanghai Laboratory Animal Center (Chinese Academy of Sciences) and housed in a pathogen-free laboratory at The First Affiliated Hospital of Zhejiang University. The procedures and protocols of the study conformed to the Guide for the Care and Use of Laboratory Animal published by the US National Institutes of Health (NIH Publication number 85-23, revised 1996) and the guidelines of the Animal Care and Use Committee of Zhejiang University.

Diabetic mice were induced by daily injection of STZ (Sigma-Aldrich, St. Louis, MO, USA) at a dose of 50 mg/kg for 5 days after a 4 h fast; control animals received the vehicle (citrate buffer; 0.05 mol/L, pH: 4.5). At the time of 0, 8, and 16 weeks after the fifth STZ injection, fasting blood glucose levels were measured in venous blood drawn from the tail by using a CONTOUR glucose meter (Bayer, Mishawaka, IN, USA). Mice with fasting plasma glucose over 13.9 mM were considered diabetic. After measurement of glucose level, diabetic mice were sacrificed after, respectively, 0, 8, and 16 weeks of diabetic duration, and their blood samples were collected. The diabetic duration was confirmed according to the process of atherosclerosis (pre-AS, labile-AS, and moderate-AS) as previously reported [13–15].

### 2.2. Glucose and Lipid Analysis

Fasting blood glucose (FBG) levels were evaluated as described above. Serum total cholesterol (TC), high-density lipoprotein cholesterol (HDL-C), low-density lipoprotein cholesterol (LDL-C), and triglyceride (TG) concentrations were determined by commercial enzymatic methods (test kits from Shanghai Rongsheng Biotech, Inc., Shanghai, China).

### 2.3. Histological Analysis

The aorta was dissected in situ from the ascending aorta to the iliac bifurcation, cleaned of peripheral fat under a dissecting microscope, and then fixed in 10% neutral formalin, embedded in paraffin, and sequentially stained with hematoxylin and eosin. Lesion areas (LA) per section were counted by taking the average of 6 sections spaced 30 *μ*m apart, beginning at the base of the aortic root. Media thickness (MT) at 10 different points of the thoracic aorta was measured and calculated. Morphometric analysis above was performed with Image-Pro Plus 6.0.

### 2.4. Cell Culture

Mouse VSMCs were isolated from thoracic aortic explants of female BALB/c mice as previously described [16, 17]. In brief, aortic explants were cultured in Dulbecco's modified Eagle's medium (DMEM; Gibco, Grand Island, NY, USA) supplemented with 10% fetal bovine serum (FBS; Gibco) and maintained at 37°C in a humidified atmosphere of 5% CO_2_ and 95% air. After 2 weeks, cells that had migrated onto the tissue culture dish were collected by trypsinization and subcultured successively. The identity of the VSMC was determined by the positive immunocytochemistry reactivity to smooth muscle specific *α*-actin. To ensure the consistency of results, passages 5–12 of VSMCs were used for experiment.

### 2.5. Proliferation Assay

VSMC proliferation was measured by the 3-[4,4-dimethylthiazol-2-yl]-2,5-diphenyltetrazolium bromide (MTT) assay. Cells were cultured in 10% FBS-DMEM on 96-well plates (5 × 10^3^ cells/well). When the VSMCs reached a 60% confluent state, the cells were rendered quiescent by incubation in DMEM containing NG and 0.1% FBS for 24 h. Next, the VSMCs were treated with different glucose concentrations (5.6, 11.1, 16.7, and 22.2 mM) for 48 h. The same concentrations of mannitol were used as an osmotic control. The cells of 96 wells were incubated with 100 *μ*L of 0.5 mg/mL MTT at 37°C for 4 h, washed with cold PBS, and lysed with 100 *μ*L of DMSO (Sigma, St. Louis, MO, USA). After the insoluble crystals were completely dissolved, the optical density of each well was immediately measured at 570 nm using an automatic microplate reader (Molecular Devices, Sunnyvale, CA).

### 2.6. Quantitative Real-Time Reverse Transcription Polymerase Chain Reaction (QRT-PCR)

Total RNA of the aortic media (VSMCs were the only cell type in this layer) or cultured VSMCs was extracted with TRIzol reagent (Invitrogen, Carlsbad, CA, USA). Complementary DNA was subsequently synthesized from RNA using the PrimeScript 1st Strand cDNA Synthesis Kit (TAKARA Biotechnology Co. Ltd., Dalian, China) following the manufacturer's instructions. cDNA was amplified afterward using the SYBR Premix Ex Taq II for Perfect Real Time (TAKARA Biotechnology Co. Ltd., Dalian, China). A housekeeping gene, *β*-actin, was used as internal control for normalizing the QRT-PCR data. Primers employed for *β*-actin and key enzymes were listed in Table 1.

### 2.7. Western Blot Analysis

Total proteins were isolated from the aortic media (VSMCs were the only cell type in this layer) or cultured VSMCs, and the procedure of western blot analysis was performed as described in our previous reports [8–10]. The expressions of key enzymes in mevalonate pathway were detected using their specific antibodies: HMGR (ab174830, Abcam, UK), P-HMGR (09-356, Millipore, USA), FPPS (ab38854, Abcam, UK), SQS (sc-136372, Santa Cruz, Japan), GGPPS (ab167168, Abcam, UK), FNT-*β* (ab74206, Abcam, UK), or GGT-1*β* (sc-18996, Santa Cruz, Japan). To ensure equal protein loading, *β*-actin was used as an endogenous control (ab8226, Abcam, UK).

### 2.8. Statistical Analysis

Values are reported as mean ± SEM. All analyses were performed with SPSS (ver. 13.0; Chicago, IL, USA). One-way analysis of variance (ANOVA) followed by Bonferroni post hoc test was used to determine significant differences between groups. Differences were considered statistically significant at a value of *P* < 0.05.

## 3. Results

### 3.1. Glucose and Lipid Content

As expected the STZ-induced mice had extremely higher levels of glucose than age-matched control groups. At 8- and 16-week diabetic groups, the levels of fasting blood glucose were separately 19.54 ± 1.39 mM and 21.04 ± 1.42 mM (both *P* < 0.01 versus age-matched control) (Table 1). However, in all groups, serum lipid levels (TC, LDL-C, HDL-C, and TG) were comparable (Table 2).

### 3.2. Atherosclerosis Development

**Figure 2:**
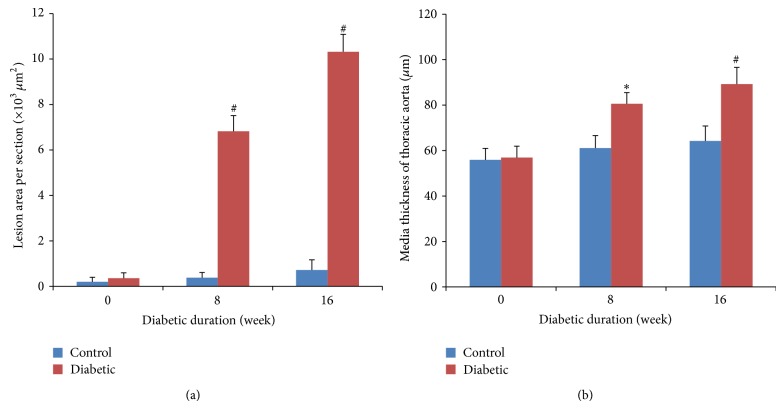
Histological analysis of aortas. (a) Lesion areas (LA) of aorta from BALB/c mice at 0-, 8-, and 16-week diabetic duration. (b) Media thickness (MT) of the thoracic aorta from BALB/c mice at 0-, 8-, and 16-week diabetic duration. Data were obtained from 6 mice for each group. Data expressed as mean ± SEM. ^∗^ *P* < 0.05 versus age-matched control mice; ^#^ *P* < 0.01 versus age-matched control mice.

The morphologic data of lesion area (LA) and media thickness (MT) are summarized in Figure 2. In our study, STZ-induced diabetic mice for 8 weeks developed mild aortic atherosclerotic plaques, with a mean LA of (6.82 ± 0.69) × 10^3^ *μ*m^2^ (*P* < 0.01 versus age-matched control). Moreover, in 16-week diabetic mice, aortic atherosclerotic plaques were moderate, with a mean LA of (10.32 ± 0.77) × 10^3^ *μ*m^2^ (*P* < 0.01 versus age-matched control). In contrast, no lesions were seen for control mice regardless of time duration. On the other hand, the MT of the thoracic aorta was increased in both 8- and 16-week diabetic mice when compared with age-matched controls.

### 3.3. Proliferation of VSMCs

**Figure 3:**
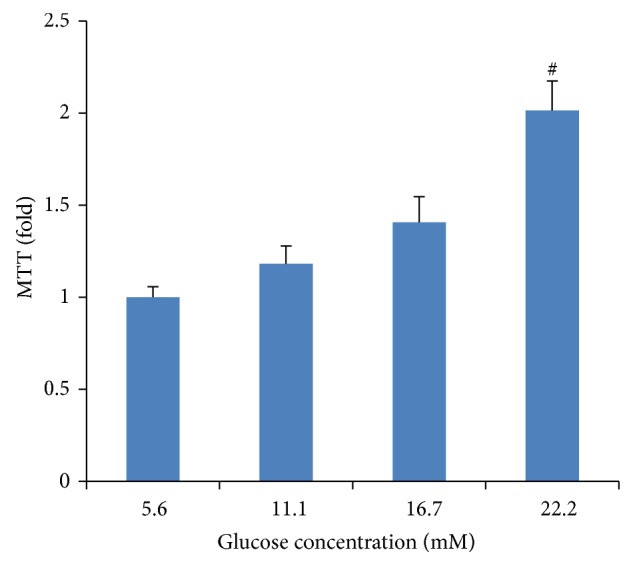
High-glucose-induced proliferation of vascular smooth muscle cells (VSMCs). VSMCs were incubated in various concentrations of glucose (5.6, 11.1, 16.7, and 22.2 mM) for 48 h. Cell proliferation was measured by an MTT assay. Mannitol was used as an osmotic control. Data expressed as mean ± SEM. ^∗^ *P* < 0.05 versus normal glucose (5.6 mM) group; ^#^ *P* < 0.01 versus normal glucose (5.6 mM) group.

Incubation of VSMCs with various concentrations of glucose (5.6, 11.1, 16.7, and 22.2 mM) resulted in an increase of VSMC proliferation in a dose-dependent manner after 48 h of treatment. This increase was not seen in the osmotic control cells treated with mannitol to maintain the same osmolarity as cell grown in a high concentration of glucose (data not shown). We found that 22.2 mM glucose significantly increased VSMC proliferation (Figure 3) compared to control cells exposed to 5.6 mM glucose (*P* < 0.01).

### 3.4. HMGR Expression Levels

**Figure 4:**
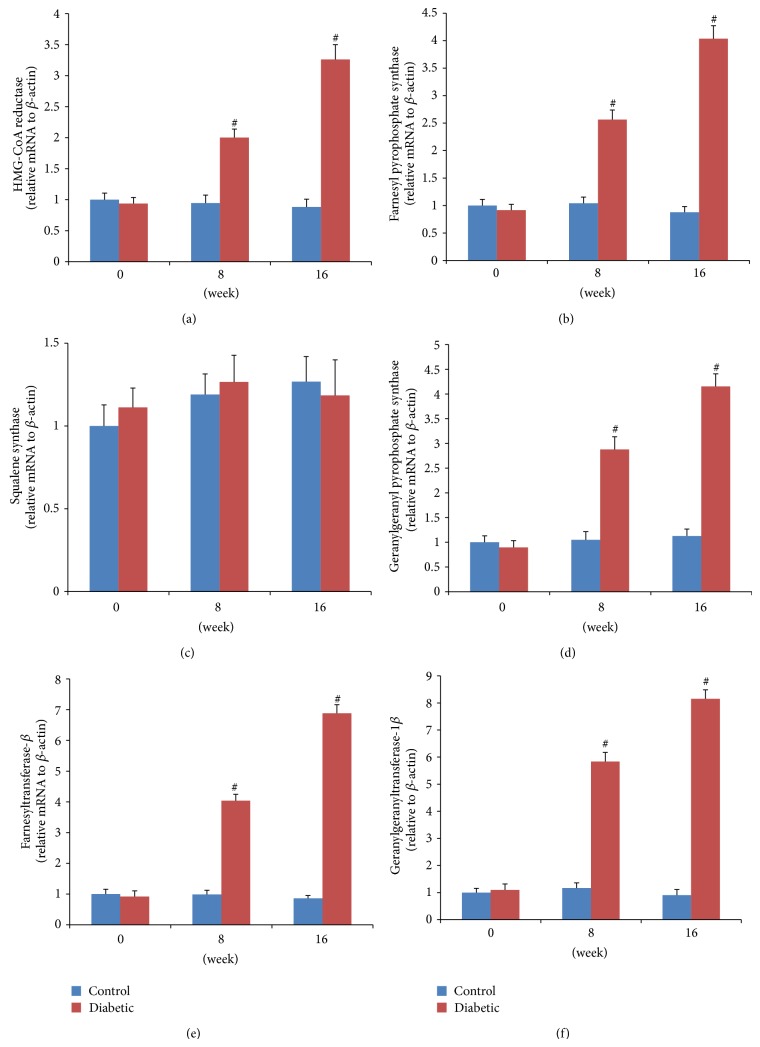
Aortic mRNA expressions of key enzymes using QRT-PCR. The relative expression level of each sample was calibrated by the comparative threshold cycle method, using *β*-actin as an endogenous control. Data are expressed as fold changes (mean ± SEM), normalized to *β*-actin mRNA expression. ^∗^ *P* < 0.05 versus age-matched control mice; ^#^ *P* < 0.01 versus age-matched control mice.

HMGR is the rate-controlling enzyme of the mevalonate pathway and is modulated through negative feedback regulation by sterols and nonsterol metabolites derived from mevalonate (Figure 1). The mRNA expressions of HMGR were significantly higher in both 8-week (mild-AS phase) and 16-week (moderate-AS phase) diabetic mice than those in age-matched control mice (Figure 4(a)). In vitro, high glucose concentrations (16.7 and 22.2 mM) significantly upregulated mRNA expression of HMGR compared to control VSMCs exposed to 5.6 mM glucose (Figure 6(a)).

**Figure 5:**
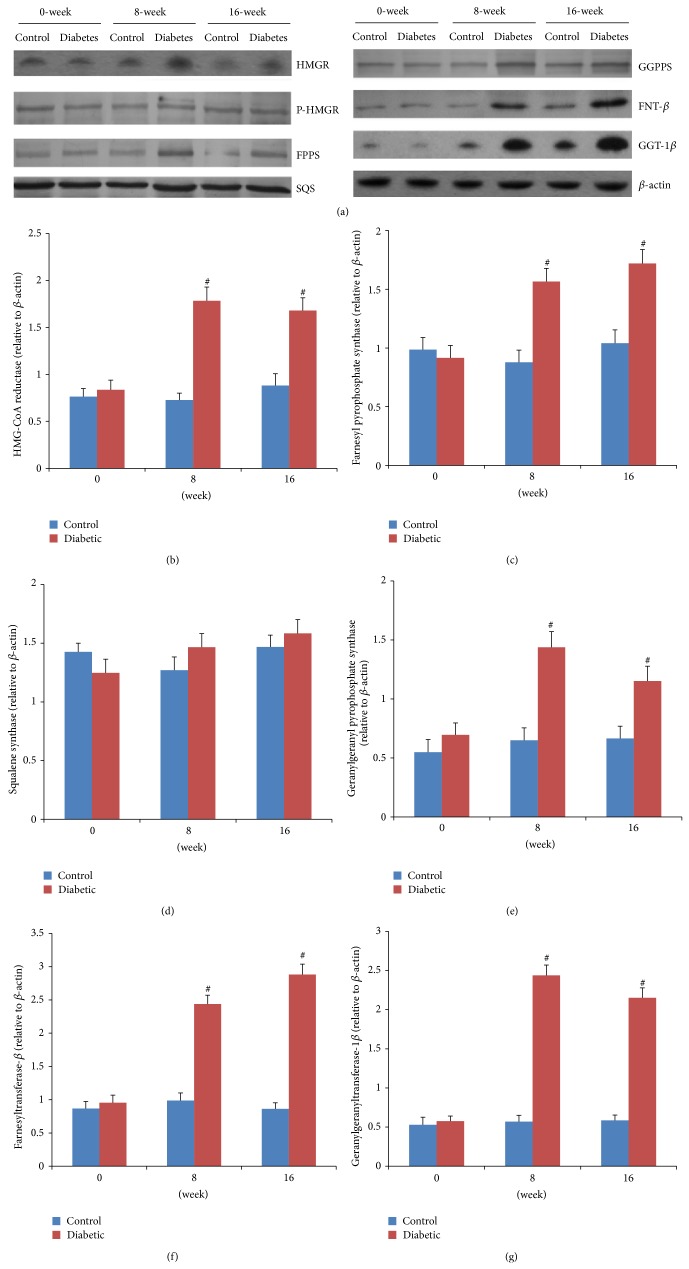
Aortic protein expressions of key enzymes by western blot. (a) Representative blots are shown. *β*-actin was used as a loading control. ((b)–(g)) The densitometric average was normalized to the values obtained from the analysis of *β*-actin as an internal control. Data expressed as mean ± SEM. ^∗^ *P* < 0.05 versus age-matched control mice; ^#^ *P* < 0.01 versus age-matched control mice. HMGR, 3-hydroxy-3-methylglutaryl-coenzyme A reductase; P-HMGR, phosphor-3-hydroxy-3-methylglutaryl-coenzyme A reductase; FPPS, farnesyl pyrophosphate synthase; SQS, squalene synthase; GGPPS, geranylgeranyl pyrophosphate synthase; FNT-*β*, farnesyltransferase-*β*; GGT-1*β*, geranylgeranyltransferase-1*β*.

**Figure 6:**
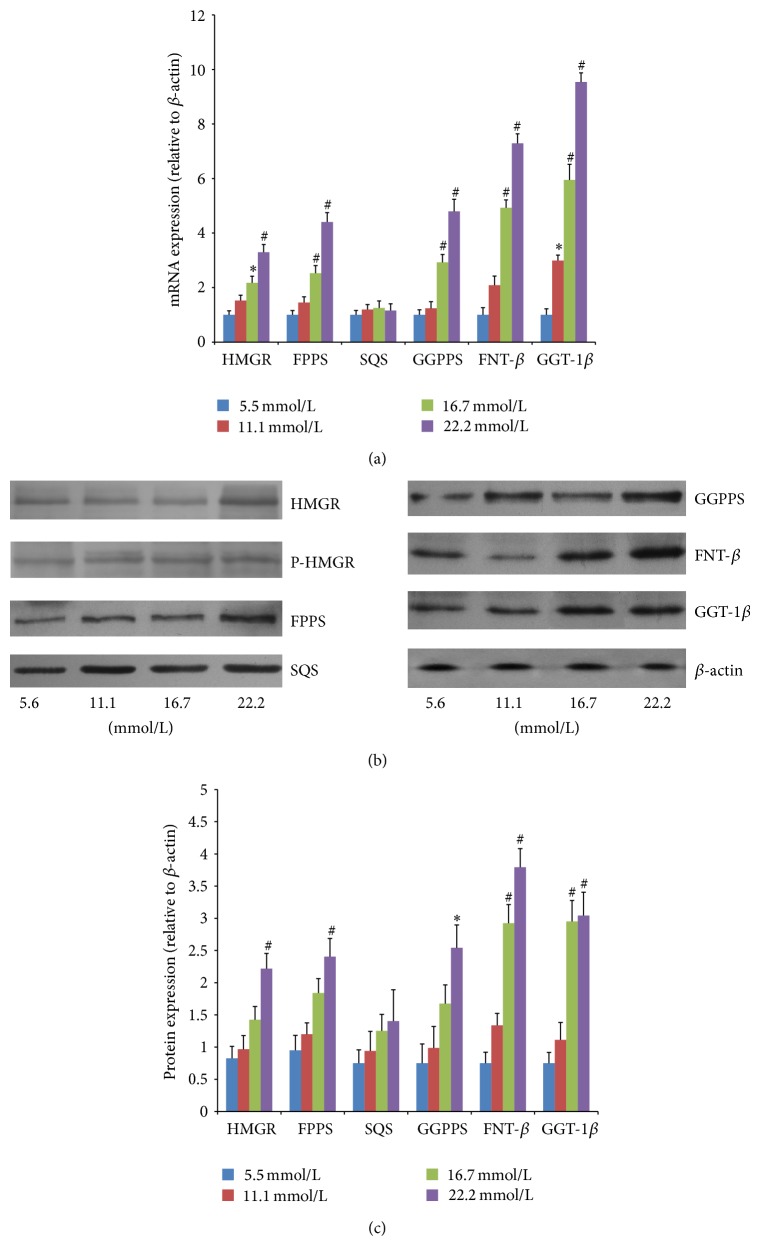
Changes of key enzymes in cultured VSMCs treated with different glucose concentrations for 48 h. (a) Gene expressions from QRT-PCR. The relative expression level of each sample was calibrated by the comparative threshold cycle method, using *β*-actin as an endogenous control. (b) Protein expressions from western blot analysis. *β*-actin was used as a loading control. Data expressed as mean ± SEM. ^∗^ *P* < 0.05 and ^#^ *P* < 0.01 versus normal glucose group (5.6 mmol/L). HMGR, 3-hydroxy-3-methylglutaryl-coenzyme A reductase; P-HMGR, phosphor-3-hydroxy-3-methylglutaryl-coenzyme A reductase; FPPS, farnesyl pyrophosphate synthase; SQS, squalene synthase; GGPPS, geranylgeranyl pyrophosphate synthase; FNT-*β*, farnesyltransferase-*β*; GGT-1*β*, geranylgeranyltransferase-1*β*.

Then, short-term regulation of HMGR is mainly ensured by phosphorylation and dephosphorylation process [18]. The phosphorylated HMGR is inactive, while the dephosphorylated one is active. In our experiment, the protein expression of HMGR in the aorta was significantly higher in both diabetic groups than the control group (Figures 5(a) and 5(b)). In contrast, the expression of P-HMGR, inactivated enzyme reversibly by phosphorylation [18], was similar in diabetes and nondiabetes mice (Figure 5(a); the densitometric average of P-HMGR was not shown). In vitro, VSMCs treated with 22.2 mM glucose remarkably induced protein expression of HMGR but not the P-HMGR (Figures 6(b) and 6(c); the densitometric average of P-HMGR was not shown).

### 3.5. FPPS Expression Levels

FPPS is a key enzyme in mevalonate pathway and directly catalyzes the formation of FPP. As seen in Figure 1, FPP is a major branching point intermediate of the mevalonate pathway that leads to several classes of essential metabolites including sterols, such as cholesterol, and nonsterols substrates for farnesylation and geranylgeranylation of proteins [19]. In both 8-week and 16-week diabetic mice, mRNA expressions of FPPS were remarkably higher than those in age-matched control groups (Figure 4(b)). Similarly, the protein expressions were also significantly higher in diabetic mice than those in control (Figure 5(c)). In cultured VSMCs, we found that 22.2 mM glucose significantly increased FPPS mRNA and protein expressions compared to control cells exposed to 5.6 mM glucose (Figure 6).

### 3.6. SQS Expression Levels

SQS represents one of the branches in mevalonate pathway, locating downstream of FPP (Figure 1). It catalyzes the synthesis of squalene from FPP, acting as the first and solely committed step towards the biosynthesis of the cholesterol, and, hence, is considered to be a potential regulatory point for sterol metabolism [20]. In our study, neither mRNA nor protein expression of SQS was different among control and two diabetic groups (Figures 4(c) and 5(d)). These findings could explain the comparable lipid levels in both control and diabetic groups. Also, after treatment with various concentrations (5.6, 11.1, 16.7, and 22.2 mM) of glucose for 48 h, VSMCs exhibited no remarkable change of SQS expression (Figure 6).

### 3.7. GGPPS, FNT-*β*, and GGT-1*β* Expression Levels

As seen in Figure 1, another branch locating downstream of FPP is nonsterol isoprenoid biosynthetic pathway, including several key enzymes of GGPPS, FNT, and GGT-1. GGPPS mediates the condensation of FPP with isopentenyl diphosphate to yield GGPP, a precursor of geranylgeranylated proteins [21]. FNT and GGT-1 are enzymes that carry out the process of prenylation in the cell [22]. They are heterodimers that share the same *α* subunit but have different *β* subunits. Thus, to assess differences of FNT and GGT-1 between diabetic and control mice, mRNA and protein expression were detected for FNT-*β* and GGT-1*β*. In our study, aortas from both 8- and 16-week diabetic duration exhibited greater mRNA and protein levels of GGPPS, FNT-*β*, and GGT-1*β* than those from age-matched controls (Figures 4(d)–4(f) and 5(e)–5(g)). In cultured VSMCs, high concentration of glucose (22.2 mM) significantly stimulated mRNA and protein expressions of GGPPS, FNT-*β*, and GGT-1*β* compared to 5.6 mM glucose treatment (Figure 6).

## 4. Discussion

Elevated levels of glucose are related to most of the complications secondary to diabetes, especially the macrovascular complication [23, 24]. The role of glucose in this complication is not completely understood and probably has a multifactorial origin. The present results show, in an experimental mouse model of atherosclerosis secondary to hyperglycemia, that there is a complicated system of enzyme expression between the sterol branch and the nonsterol isoprenoid branch signaling pathway in STZ-induced diabetic aortas.

A clear relationship between diabetes and cardiovascular disease has been established for decades. Despite this, the mechanisms by which diabetes contributes to plaque formation remain in question. Pathophysiologically, the lesions of atherosclerosis represent a series of highly specific cellular and molecular responses. Atherosclerosis can develop in response to endothelial cell (EC) injury caused by hyperglycemia. After initial injury, different cell types including EC, platelets, and inflammatory cells release mediators, such as growth factors and cytokines that induce multiple effects. These growth factors and cytokines will promote the changes of VSMC from the quiescent contractile state to the synthetic proliferative state, exaggerating VSMC proliferation and migration [4, 5, 25]. It has been widely accepted that VSMC proliferation is an important event in the pathophysiological course of atherosclerosis and attracts more attention of the researchers. In our study, BALB/c mice made diabetic by STZ treatment exhibited different levels of plaque formation, from mild to moderate, due to different diabetic duration, which is in agreement with most of the previous studies [26, 27].

The signaling pathways by which changes in glucose metabolism modulate VSMC proliferation are poorly understood. Numerous molecular and cellular studies have demonstrated that small GTP-binding proteins, consisting of the Ras and Rho family (RhoA and Rac1), participate in the VSMC mitogenic machinery triggered by hyperglycemia. Indeed, extracellular signal-regulated protein kinases (ERKs) (also known as the 42 and 44 kDa isoforms (ERK1/2) pathway) are typically initiated by Ras, which can be stimulated by inflammatory cytokines from high-glucose injured EC [28–30]. The activated ERK1/2 proteins eventually lead to VSMC proliferation and intimal thickening [28, 29, 31]. Moreover, RhoA has been reported to be abundantly expressed in cardiovascular tissues. RhoA and Rho kinase may also be involved in hyperglycemia-induced cell growth and serum response element- (SRE-) dependent c-fos gene expression in rat aortic smooth muscle cells [32]. Furthermore, several studies have identified the key role for Rac1 GTPase in the regulation of high-glucose- and diabetes-induced VSMC proliferation [33, 34]. Zhu and coworkers reported that high-glucose treatment resulted in phosphorylation and membrane translocation of Rac1, p47phox, and p67phox subunits, leading to NADPH oxidase activation and reactive oxygen species (ROS) production, subsequently promoting VSMC proliferation and vascular dysfunction [34].

As stated above, small GTPases (including Ras, RhoA, and Rac1) appear to exert multiple effects on high-glucose-induced VSMC proliferation. For their part, the membrane localization and activation of Ras, RhoA, and Rac1 depend on the process of isoprenylation [35–37]. Ras translocation from the cytoplasm to the plasma membrane is dependent on farnesylation by FNT with FPP, whereas translocation of RhoA and Rac1 is dependent on geranylgeranylation by GGT-1 with GGPP [22]. In our study, we found increased expression of HMGR and FPPS and similar SQS in the aorta from STZ-induced diabetic mice. As shown in Figure 1, HMGR and FPPS are two key enzymes, locating upstream of mevalonate pathway, and directly or indirectly catalyze the synthesis of isoprenoid intermediates (FPP and GGPP). SQS represents one of the branches of mevalonate pathway, locating downstream of FPP. Our findings support the viewpoint that pathologic VSMC proliferation in diabetic aorta may be caused by toxic accumulation of isoprenoid intermediates, including FPP and GGPP, which leads to excessive small GTPase activation.

Theoretically, both HMGR inhibitors (statins) and FPPS inhibitors (nitrogen-containing bisphosphonate, N-BP) may inhibit the synthesis of isoprenoid intermediates, suppress the activation of small GTPase, attenuate the VSMC proliferation, and finally slow the progression of atherosclerosis induced by diabetes. Indeed, the reality is more complex. A wealth of both experimental and clinical evidence reveals the beneficial effects of statins on atherosclerotic progression with diabetes [38, 39], including inhibition of VSMC proliferation and migration [40, 41]. However, large trials suggested an increased incidence of new onset diabetes with long-term use of statins [42–44]. No exact mechanism has been established. Some investigators have proposed that statins may cause an immune response that interferes with insulin signaling [45]. On the other hand, N-BPs, which are extensively used in bone-related disorders [46], have been reported to inhibit the development of experimental atherosclerosis without altering serum lipid profile [47, 48]. In vitro study showed that the antiatherosclerotic effect of N-BPs might be due to inhibition of VSMCs, at least for zoledronate [49]. However, current evidence is not conclusive and further research is necessary to confirm these actions in the clinical setting.

In our study, beside HMGR and FPPS, the expressions of GGPPS, FNT, and GGT-1 were also remarkably upregulated in diabetic aortas. In cultured VSMCs, high glucose induced cell proliferation, accompanying upregulated HMGR, FPPS, GGPPS, FNT, and GGT-1. All these results showed that these enzymes of mevalonate pathway were upregulated in atherosclerotic aortas from diabetes, and the changes may mainly focus on the vascular media-VSMCs. Therefore, we inferred that these enzymes may play important roles in diabetic atherosclerosis, especially in VSMC proliferation. Then, GGPPS, FNT, and GGT-1 locating downstream of FPP to synthesize nonsterol isoprenoid might be regarded as candidate targets for antiatherosclerosis intervention. Further study is necessary.

In conclusion, our work provided the evidence that alteration of the expression of key enzymes in the mevalonate pathway in aorta from diabetes mice is implicated in the atherogenic process, probably through activation of small GTPases and inhibition of VSMC proliferation. Although there are many essential differences between experimental and clinical studies, our study indicates that interfering with the upregulation of key enzymes and biosynthesis of isoprenoids in the mevalonate pathway may open the field for novel therapeutic indications and give many possible therapeutic targets for diabetic macrovascular diseases in the future.

## Figures and Tables

**Table 1:** Glucose and lipid analysis at 0-, 8-, and 16-week diabetic duration in BALB/c mice.

Week	FBG (mM)	TC (mM)	HDL-C (mM)	LDL-C (mM)	TG (mM)
Control mice					
0 (*n* = 6)	7.82 ± 0.70	1.82 ± 0.07	0.93 ± 0.06	0.48 ± 0.06	1.31 ± 0.05
8 (*n* = 6)	7.66 ± 0.62	1.88 ± 0.07	0.86 ± 0.07	0.44 ± 0.06	1.30 ± 0.05
16 (*n* = 6)	8.40 ± 0.61	1.90 ± 0.08	0.93 ± 0.06	0.45 ± 0.06	1.25 ± 0.05
Diabetic mice					
0 (*n* = 6)	8.20 ± 0.63	1.93 ± 0.09	0.85 ± 0.06	0.47 ± 0.05	1.26 ± 0.06
8 (*n* = 6)	19.54 ± 1.39^#^	1.91 ± 0.08	0.84 ± 0.05	0.48 ± 0.07	1.35 ± 0.06
16 (*n* = 6)	21.04 ± 1.42^#^	1.96 ± 0.08	0.77 ± 0.06	0.53 ± 0.07	1.40 ± 0.05

Data expressed as mean ± SEM.

^#^ *P* < 0.01 versus age-matched control mice.

FBG, fasting blood glucose; TC, total cholesterol; HDL-C, high-density lipoprotein cholesterol; LDL-C, low-density lipoprotein cholesterol; TG, triglyceride.

**Table 2:** Sequences of forward and reverse primer.

cDNA	Forward primer	Reverse primer
HMGR	5-GAATGCAGAGAAAGGTGCAA-3	5-CACCACGTTCATGAGTTTCC-3
FPPS	5-GCACTGACATCCAGGACAAC-3	5-TAAGATCTGGCGCTGTTGAG-3
SQS	5-CAAGCAGGTCATCTCCAAGA-3	5-GCAGCCAAGAGCATGATAAA-3
GGPPS	5-TGGAACTTCATCAGGGACAA-3	5-CCTGTCTTCTGCAACACCAT-3
FNT-*β*	5-TGGCAGTAATCCTGGAACTG-3	5-GAGCTCGCTGTGACTGTAGC-3
GGT-1*β*	5-GCTAAATGGCCAGACAGTCA-3	5-CAAATCCCACTCTCCTCCAT-3
*β*-actin	5-CTCCATCCTGGCCTCGCTGT-3	5-GCTGTCACCTTCACCGTT-3

HMGR, 3-hydroxy-3-methylglutaryl-coenzyme A reductase; FPPS, farnesyl pyrophosphate synthase; SQS, squalene synthase; GGPPS, geranylgeranyl pyrophosphate synthase; FNT-*β*, farnesyltransferase-*β*; GGT-1*β*, geranylgeranyltransferase-1*β*.
